# Use of iron supplements in children aged 1-2 years with iron deficiency anemia: A cross-sectional study

**DOI:** 10.12669/pjms.315.7334

**Published:** 2015

**Authors:** Handan Atsiz Sezik, Huseyin Can, Mehmet Ali Kurnaz, Mine Tuna, Zeynep Ay

**Affiliations:** 1 Dr. Handan Atsiz Sezik, MD. Specialist, Katip Celebi University Ataturk Education and Research Hospital, Family Medicine Clinic, Izmir/Turkey; 2 Dr. Huseyin Can, MD. Assistant Professor, Katip Celebi University Ataturk Education and Research Hospital, Family Medicine Clinic, Izmir/Turkey; 3 Dr. Mehmet Ali Kurnaz, MD. Specialist, Katip Celebi University Ataturk Education and Research Hospital, Family Medicine Clinic, Izmir/Turkey; 4 Dr. Mine Tuna, MD. Specialist, Katip Celebi University Ataturk Education and Research Hospital, Family Medicine Clinic, Izmir/Turkey; 5 Dr. Zeynep Ay, MD. Katip Celebi University Ataturk Education and Research Hospital, Family Medicine Clinic, Izmir/Turkey

**Keywords:** Anemia, Family medicine, Iron deficiency, Iron supplementation

## Abstract

**Objectives:**

Iron deficiency (ID) is the most common nutritional problem in the world and is the most common cause of childhood anemia. In this study, our aim was to find out about the state of usage of iron preparation, which is distributed free of charge by the Ministry of Health, for the infants between 4-12 months in our country, as well as detecting the awareness degree of families those who are informed about iron-deficiency anemia (IDA), prophylaxis of the drug and to determine the drug’s effectiveness.

**Methods:**

It was a cross-sectional survey. The laboratory values from the files of the children aged 1-2 those who visited our hospital’s department of pediatrics, between January 2010 to August 2013, were collected. The survey included families who have children diagnosed with IDA. Questions included about families’ sociodemographic characteristics, the state of the usage of the iron drug, how much information received in terms of the side effects- consumption period and dosage.

**Results:**

A total of 139 children were enrolled in our study. While 77.7% of the families who participated stated that (n = 108) iron medicine was prescribed other 43.2% of families stated (n = 60) was prescribed and they were informed about iron pills and IDA. 25.9% of families had received information about drug’s side effects, 74.8% of them had information about period of consumption and 77.7% said they were given information about the drug dose. The average duration of use of iron medicine was 6.98±4.52 (min: 1, max: 24) months. It has been noted that; parent’s education level, mother’s occupation, child’s gender, how the child was born and receiving information about how to use the medicine had no effects on usage of the drug in children. Nevertheless, it has been noticed that, when the families were given proper information the drug use increased and the patients compliance with medications also increased.

**Conclusion:**

We believe that, due to frequent diagnosis of anemia in children, prophylaxis iron supplementation should be continued for up to the age of two years.

## INTRODUCTION

Iron is an element, which is found in natural food abundantly. However even then iron deficiency is the most common nutritional problem in the world and the most common cause of childhood anemia.^1^,^2^ The efforts are being made to cure negative effects of IDA but is still very serious public health problem affecting a large number of children and women in developing countries. It is the only nutritional deficiency which is also significantly prevalent in industrialized countries. It is essential that every newborn is provided with appropriate environment, housing conditions, a balanced diet and proper education.

Anaemia is a condition in which the number and size of red blood cells, or the haemoglobin concentration, falls below an established cut-off value, consequently impairing the capacity of the blood to transport oxygen around the body. Anaemia is an indicator of both poor nutrition and poor health.^3^

Iron deficiency anaemia is seen in young children and is common in women. The most common cause of IDA is rapid growth of increased iron requirements due to insufficient iron intake and blood loss. The most important cause of ID is the imbalance between dietary iron and iron requirements necessary for metabolic functions. The amount of iron supplied from erythrocytes is destroyed up to 70% due to the rapid growth in children. During this period, 30% of the dietary intake has to be through a diet for the vital functions of the body and erythropoiesis, Insufficiencies of a diet result in more ID.^4^

IDA affects growth and development in children and decreases resistance against infections, psychomotor and cognitive developments are also delayed while intelligence level is affected adversely.^1^,^5^ Children under two years of age with ID are observed to have adaptation and balance problems as well as showing anti-social and timid behavior. If the ID is not prevented, it also affects the productivity of the children.

According to WHO’s worldwide data between 1993-2005, preschool children are especially affected and the prevalence of anemia in this group was found to be 47.4% or 293 million children are identified as affected.^6^ Today, nearly 600 million preschool and school age children are anemic.^7^ When the data is analyzed, the highest prevalence of anemia is in Africa (67.6%) followed by Asia (65.5%). The WHO public health IDA symptom categories based on the prevalence of ≤ 4.9% means no public health problem, and 5 to 19.9% mild public health problem, and 20 to 39.9% moderate public health problem, ≥% 40 is defined as a severe public health problem. According to these values all around the world; 69 countries are classified as severe, 81 moderate and 40 countries are as having mild problem of iron deficiency anemia while two countries appears to have no problems in terms of IDA.^6^

Economic analysis shows that iron deficiency anemia can be prevented with low costs. WHO recommends prophylactic iron preparations for 6-23 month-old babies when the diet does not contain nutrient-enriched dietary iron or prophylactic anemia prevalence is over 40%.^1^ When the development is significant and at the time of increased necessity, iron supplement should be given as a treatment when anemia is confirmed as prophylactic.

In our country under the name of “Iron-like Turkey” project 4 -12 month-old infants are being given prophylactic iron pills free of charge since 2004 by the Ministry of Health for primary health care purposes. Iron medication, which is supplied by The Ministry of Health, is prescribed and distributed free by family physician organizations where basic health care services are provided. In this project, the infants aged 4-12 months, are given 10 mg/day iron medication as a single dose during the six months duration of treatment again free of charge. This drug is in the form of oral drops. One drop of the dispensed drug is equivalent to 2.5 mg of elemental iron. In this project it is aimed to inform family physician about iron-deficiency anemia, the drug usage and dosage, side effects and its benefits. In Turkey, this project is still in progress and prophylactic iron medication is distributed to families.

In this study our aim was to determine iron drug distribution-consumption and the degree of information families received about IDA and the side effects consumption duration-dosage of drug prophylaxis as well as to determine the drug’s effectiveness, which is distributed in basic health care centers by family physician which started in 2004 by the Ministry of Health under the project name of “Iron-like Turkey”.

## METHODS

This is a cross-sectional study which was approved by the ethics committee of the hospital. In the first part of our study the files of patients, those who came to Izmir Katip Celebi University Atatürk Educational and Research Hospital Department of Pediatrics between 01.01.2010-01.08.2013, were analyzed.

The values of Hemoglobin (Hb), Hematocrit (Hct), red blood cell (RBC), Mean corpuscular volume (MCV), red blood cell distribution width (RDW), Iron (Fe) total iron binding capacity (TIBC), ferritin were examined from the files of 1-2 year age children who were diagnosed with preliminary anemia. Patients diagnosed with IDA were the main focus of our study. In the second part of our study families of children with IDA were surveyed with a questionnaire that was pre-prepared. In all twenty eight questions were asked to the families, 14 of these questions were to determine socio demographic characteristics of the families, while remaining 14 were to determine the degree of information families received about IDA and the side effects-consumption duration-dosage of drug prophylaxis. Families’ were asked to give verbal consent over the telephone. Families without phone numbers in hospital’s automation system, or those who could not be reached by phone or refused to participate in our survey were excluded.

Results were analyzed using SPSS version 16. Independent t-test was used for data analysis. Chi-square test was applied for group comparisons. p value of <0.05 was considered statistically significant.

## RESULTS

Two hundred sixty one children between 1-2 years of age were identified with IDA according to laboratory values. When families without phone numbers in hospital’s automation system, or those who could not be reached by phone or refused to participate were excluded. Hence 139 children were finally included in the study. It consisted of 79 male. One hundred thirty eight (138) were breastfed after birth, 104 mothers who participated in the study were housewives, 43.2% (n = 60) received high school or higher education, and the average maternal age was 30.44 ± 5.29 years.

Ninety seven mothers (69.8%) were diagnosed with anemia during pregnancy, while 108 mothers (77.7%) said that they consumed iron supplement during pregnancy. Socio demographic characteristics of the participants are shown in Table-I. About 77.7% of the families who participated in the study (n = 108) were given the iron medicine, while 43.2% (n = 60) stated that they were informed about iron pills and IDA. About 25.9% of families stated that they had received information about drug’s side effects, 74.8% about duration of consumption, and 77.7% said that they were given information about the drug dose. The amount of iron medicine distributed by family physician and the answers given by the families about IDA is shown in Table-II. About 76.3% of families (n = 106) stated that they used iron drugs given by family physician. Those who did not use the medicine (23.7%; n = 33) gave the reasons such as intolerance of the child, negligence and drug’s side effects. The average duration of use of iron medicine was 6.98 ± 4.52 (min: 1, max: 24) months.

**Table-I:** Sociodemographic characteristics of the families and children.

		No. (n)	Rate (%)
Sex	Male	79	56.8
	Female	60	43.2
Mode of Delivery	Natural childbirth	58	41.7
	Cesarean section	81	58.3
Medical problem	Yes	33	23.7
	No	106	76.3
Mothers’ job	Housewife	104	74.8
	Employee	5	3.6
	Official	16	11.5
	Self-employed	14	10.1
Mothers’ education	Illiterate	4	2.9
	Literacy	10	7.2
	Primary school	44	31.7
	Junior high school	21	15.1
	High school	35	25.2
	University	25	18.0
Fathers’ education	Illiterate	1	0.7
	Literacy	4	2.9
	Primary school	33	23.7
	Junior high school	24	17.3
	High school	41	29.5
	University	36	25.9
Number of Children in the Family	one	41	29.5
	two	71	51.1
	three	18	12.9
	four	9	6.5

**Table-II:** The answers given by the families about IDA.

		n	%
Have you been informed about the iron deficiency anemia and iron medicine?	Yes	60	43.2
	No	77	55.4
Have you recurred medication?	Other*	2	1.4
	Yes	108	77.7
	No	26	18.7
Have you been informed about the adverse effects of the drug’s?	Other*	5	3.6
	Yes	36	25.9
	No	103	74.1
Have you been informed about the duration of drug use?	Yes	104	74.8
	No	35	25.2
Have you been informed about of the drug dose?	Yes	108	77.7
	No	31	22.3

* Other: Children followed up by a pediatric physician.

We found that, there was no relation between the use of iron medication, which is prescribed by a family physician, and parent’s education level, child’s sex, receiving information about the side effects of the medication. It is understood that housewives used more iron medication in comparison to other mothers. It was also noted that, families who were informed about the treatment of IDA, duration of consumption and about the dosage had increased usage of iron medication (Table-III).

**Table-III:** Comparison of the use of the iron drugs given to the families and sociodemographic status and percentages of informed families.

		Status of drug use		p
		Yes n (%)	No n (%)	
Mothers’ education	Below High school	62 (78.5)	17 (21.5)	0.480
	Above High school	44 (73.3)	16 (26.7)	
Fathers’ education	Below High school	46 (74.2)	16 (25.8)	0.608
	Above High school	60 (77.9)	17 (22.1)	
Mothers’ job	Housewife	75 (72.1)	29 (27.9)	0.048
	Other	31 (88.6)	4 (11.4)	
Childs’ sex	Female	47 (78.3)	13 (21.7)	0.616
	Male	59 (74.7)	20 (25.3)	
Have you been informed about the iron deficiency anemia and iron medicine?	Yes	53 (88.3)	7 (11.7)	0.008
	No	51 (66.2)	26 (33.8)	
	Other*	2 (100.0)	0 (0.0)	
Have you been informed about the side effects of the drug’s?	Yes	31 (86.1)	5 (13.9)	0.107
	No	75 (72.8)	28 (27.2)	
Have you been informed about the duration of drug use?	Yes	97 (93.3)	7 (6.7)	0.001
	No	9 (25.7)	26 (74.3)	
Have you been informed about of the drug dose?	Yes	101 (93.5)	7 (6.5)	0.001
	No	5 (16.1)	26 (83.9)	
			

* Other: Children followed up by a pediatric physician.

## DISCUSSION

IDA is currently the biggest global nutritional problem. According to WHO the prevalence of anemia in children approximately one year of age is above 40% or the diet does not include foods fortified with iron, supplements of iron at a dosage of 2 mg / kg of body weight per day should be given to all children between 6 and 23 months of age.^1^

M. McDonagh et al. stated that in their reviews of majority of routine studies of iron prophylaxis among the children 6-24 months, iron deficiency has no effect on weight loss, height, head circumference. They also stated that the clinical findings of test scores are not clear enough for developmental progress. In this review, when the levels of iron prophylaxis on IDA- hemoglobin-ferritine were assessed; in 5 studies, there were significant benefits, while there was no difference between the two study groups. About nine studies which compared the levels of hemoglobin and ferretin respectively were found to be questionable. This review suggested that the supplement of iron should be received from solid food until it reaches the sufficient level.^8^

Pasrich SR et al in there review stated that most of the researches indicate that daily supplementation of iron increases the hemoglobin levels while reducing the effects of anemia and iron deficiency incidence. However, the effects on development-growth is unclear. The supplementation of iron was claimed to be more than three months.^9^

In our study, even though routine iron supplementation was used by 78% and treatment continued for seven months, there children were still diagnosed to have anemia. Sachdev H et al. in their review in 2005 stated that “Iron supplementation improves mental development score modestly” and this effect is seen in children over 7 years of age with initial anemia. This review, did not prove convincingly if iron supplementation had any effects on motor-mental development of children under 27 months of age.^10^

Gera T et al. in their review in 2012 stated that when iron supplemented food is consumed, levels of hemoglobin, ferritin and other biomarkers increase significantly. However, there is no convincing evidence on motor mental development. In addition, this review also noted that in future studies iron content and bioavailabity should be increased in the products. In addition new strategies should be developed to increase the children’s mental development.^11^ Iannotti LL et al. state that iron supplement increases hemoglobin concentration for children with anemia or iron-deficiency anemia. They further stated that long-term, low-dose iron supplement improves cognitive and motor development.^12^

Yalcin SS et al. in their study showed that short term iron supplementation has no effects on changing mental test scores of the healthy children, because of the IDA’s high prevalence and the negative impact on the development associated with it, the need for the prophylaxis should be emphasized rather than the treatment.^13^ Wang B et al. stated in their review that in a 30-day follow-up of the children those who were given iron therapy because of the IDA there was no convincing evidence to show any effect on psychomotor development or cognitive functioning. Hence there is a need for long-term randomized controlled trials.^14^

Various studies mentioned above discuss whether there is a need for iron supplement during the childhood in terms of mental and motor development. We believe that since mental and motor development of the children is a situation that cannot be disregarded, iron should be given at least until the age of two years as a prophylactic medicine or as food which is enriched with iron.

In our study, although starting month is appropriate to start supplementary food for infants, because of high diagnosis of IDA, supplementary food should be enriched with iron. In a study conducted in Egypt, especially in rural areas where the IDA is more prevalent, it is necessary to advice the use of iron-enriched food by health care providers and prophylactic iron supplementation which should be given to all infants between 6-23 months.^15^ American Academy of Pediatrics Committee on Nutrition has reported that iron deficiency anemia can be prevented by fortification of infant formulas. Study has shown that iron deficiency anemia in the first year of life decreased from 20% to 3% by 10-12mg/L iron fortification of infant formulas between 1970-80.^16^

Jaber L. in one of his study gave standard information about diet enriched with iron to the mothers in the control group, while giving detailed information to the mothers in the intervention group as well as encouraging them to give complex polymaltos iron. In this study, “there was no effect of infant or parental background factors on rate of anaemia. Frequency of anaemia was lower in infants who received ≥6 months of iron medication according to mothers’ reports, and in infants breastfed for ≥6 months”.^17^ In our study, we also found that families who received information about IDA and iron medicine and duration of consumption, used the iron medicine more.

## CONCLUSIONS

Providing information to families with regard to the importance of iron deficiency, how to consume, duration and dosage of iron preparations, the biggest problem of adaptation, of iron supplementation program can be solved. Furthermore, because of the frequent diagnosis of anemia in children, we believe that despite the iron supplementation, iron prophylaxis should also be continued for up to the age of two years.
